# A Leiomyoma of The Liver

**DOI:** 10.1155/1989/45978

**Published:** 1989

**Authors:** M. J. Hollands, R. Jaworski, K. P. Wong, J. M. Little

**Affiliations:** Department of Surgery Westmead Hospital Westmead NSW 2145 Australia


*HPB Surgery* 1989, Vol. 3, pp. 337–343
Reprints available directly from the publisher
Photocopying permitted by licence only
© 1989 Harwood Academic Publishers GmbH
Printed in Great Britain

### CASE REPORT
### A LEIOMYOMA OF THE LIVER

M. J. HOLLANDS, R. JAWORSKI, K. P. WONG and J. M. LITTLE

*Department of Surgery, Westmead Hospital, Westmead, NSW 2145, Australia*

*(Received 15 September 1988)*

KEY WORDS: Leiomyoma, liver surgery.

### INTRODUCTION

In the following case a rare benign hepatic tumour is reported. This tumour presented as an apparently malignant lesion and its benign nature was not confirmed until a left hepatic lobectomy had been performed.

### CASE REPORT

A seventeen year old Turkish born male school student presented with a four year history of recurrent abdominal pain. The pain was epigastric in distribution and relieved by non-steroidal anti-inflammatory drugs. He had not lost weight and there were no other associated abdominal symptoms. On examination the liver edge was palpable 12 cm below the costal margin in the mid-clavicular line. There were no other findings on physical examination.

Liver function tests were normal. There was no evidence of previous hepatitis B virus infection and CEA (carcinoembryonic antigen) and AFP (alpha fetoprotein) levels were normal.

An ultrasound examination was performed which showed a hypoechoic poorly defined solid mass in the left lobe of the liver. A subsequent CT scan confirmed the presence of the mass which was enhanced by the injection of contrast medium. A provisional diagnosis of a haemangioma of the liver was made. A subsequent blood pool scan did not confirm this suspicion as there was reduced tracer accumulation in the region of the left lobe of the liver. It was decided to proceed to arteriography. This showed a vascular lesion strongly suggestive of a hepatoma (Figure 1). Venous phase pictures showed that the left branch of the portal vein had been occluded by the tumour (Figure 2). An inferior vena cavagram was then performed and this showed that the left hepatic vein was also occluded with only a thin stream of contrast seen. The hepatic vein also seemed to communicate directly with the portal venous system. Fine needle aspiration cytology of this lesion showed smooth muscle cells but there was no evidence of hepatocellular carcinoma. The origin of the cells may have been either stromal or from a smooth muscle tumour.

A laparotomy was performed nine days after admission. Inspection and palpation of the gastrointestinal tract did not reveal a potential primary site. A large tumour was found in the left lobe of the liver with intense fibrous reaction around it. The tumour appeared to extend into the falciform ligament and apparently involved the root of the left hepatic vein. Further fine needle aspiration cytology was performed and once again smooth muscle cells were seen. Frozen section suggested that the

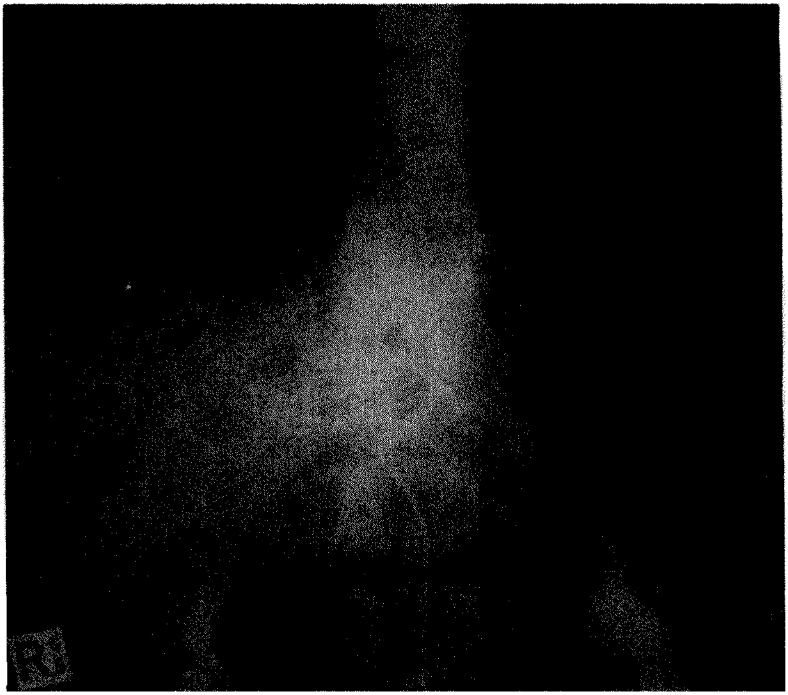

**Figure 1** Hepatic Arteriogram.

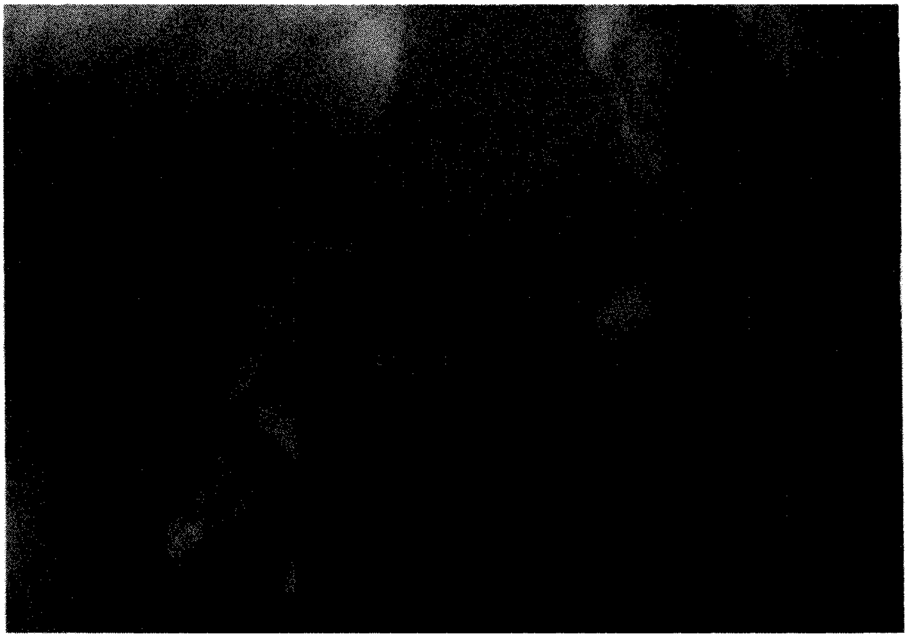

**Figure 2** Venous phase films showing occlusion of the left branch of the portal vein.

tumour was indeed a leiomyoma. A left hepatic lobectomy was then performed. Parts of segments 1 and 4 had to be preserved as it was impossible to dissect the dense fibrous reaction from the inferior vena cava.

The resected specimen consisted of the left lobe of the liver and measured 120 × 100 × 80 mm and weighed 403 grams. Located within the substance of the liver was a lobulated white tumour which had a whorled appearance on cut section (Figure 3).

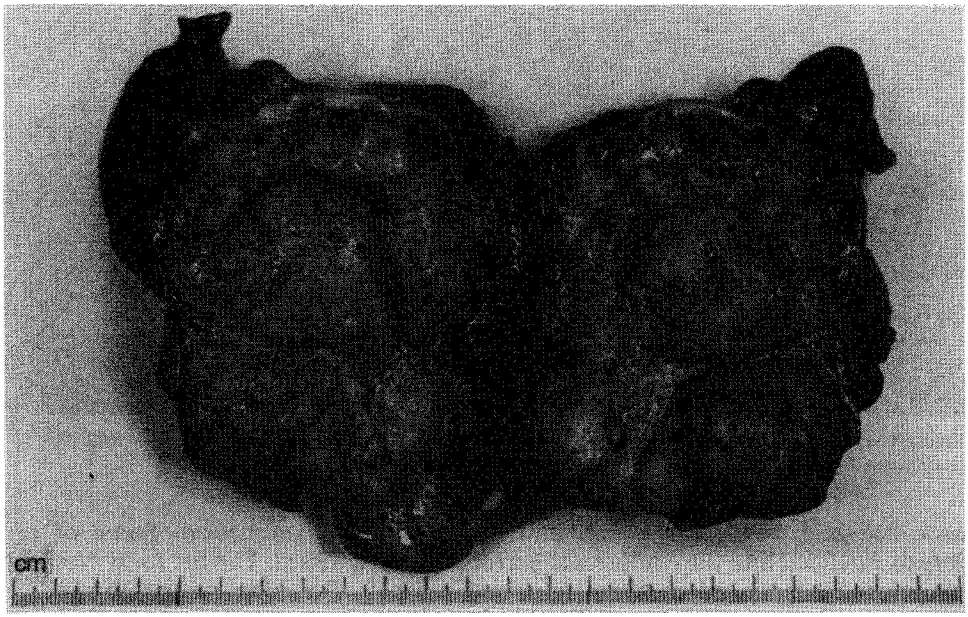

**Figure 3** Macroscopic appearance of resected liver lesion.

The tumour measured approximately 85 × 90 × 80 mm. It was almost totally enclosed within the liver parenchyma, although a nodule of tumour 30 mm in its maximum dimension projected into the hilum. At one edge of the tumour the obstructed left hepatic duct was present and this contained mucinous material. The hilum of the liver contained a number of enlarged lymph nodes. There was no evidence of tumour within the lymph nodes or within the hilar blood vessels.

Histologically the tumour consisted of a proliferation of interlacing bundles of spindle cells. The tumour cells showed elongated "cigar" shaped nuclei as well as projections of eosinophillic cytoplasm (Figure 4). There were areas of collagenisation as well as areas of myxoid change. Numerous small blood vessels were seen coursing throughout the substance of the tumour. No significant mitotic activity was seen. Formalin fixed paraffin embedded tissue sections were stained for the intermediate filament desmin using the immunoperoxidase technique. The tumour cells showed strongly positive staining. Moreover, ultrastructural examination by transmission electron microscopy on formalin fixed tissue showed typical ultrastructural features of smooth muscle differentiation. The histological and ultrastructural features were those of a leiomyoma.

Around the periphery of the tumour there was zone of dense fibrosis with

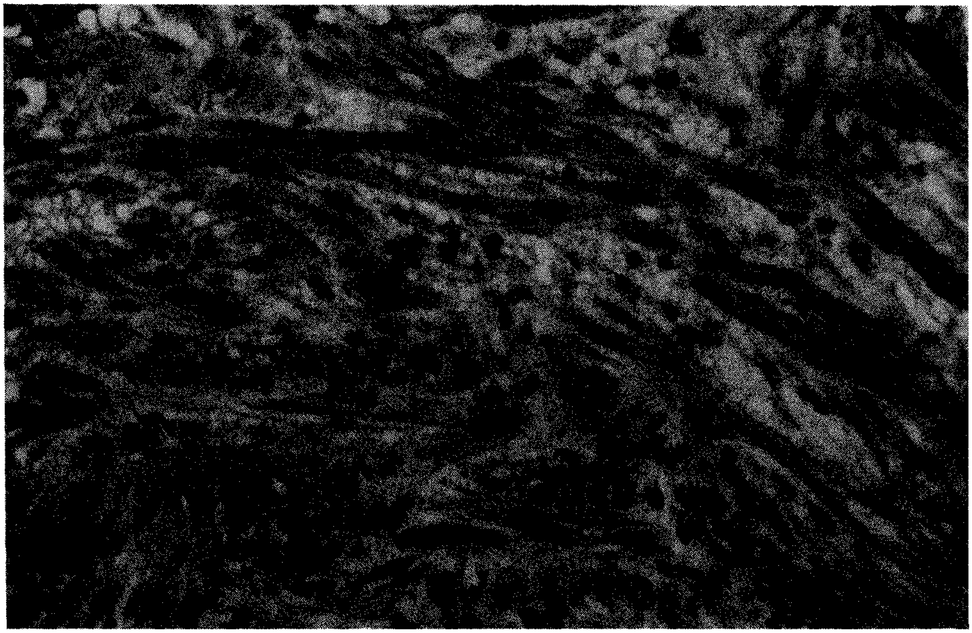

**Figure 4** Microscopic appearance of the tumour showing interlacing bundles of spindle cells with cigar shaped nuclei (magnification: 500 x).

associated bile duct proliferation and mild chronic inflammation. Some of this fibrosis extended into the compressed surrounding liver tissue. Further away from the tumour the hepatic parenchyma appeared normal. The hilar vessels showed no remarkable abnormalities. Two hilar lymph nodes showed reactive changes as well as changes consistent with lymphatic obstruction.

The patient has now been followed up for twelve months without evidence of a possible occult primary or recurrence in the liver.

### DISCUSSION

Benign tumours of the liver, with the exception of haemangiomas, are rare conditions and have been adequately reviewed[1,2,3,4]. Three cases of leiomyoma in the liver have previously been reported[5,6,7]. This is a rare tumour and perhaps because of its rarity is worthy of publication. The importance of this case however is not just its rarity but the way in which it was able to masquerade as a malignant tumour of the liver.

Clinically, this case has a number of features suggestive of benign disease. The patient was young, male, did not use androgen supplements and did not have cirrhosis. Neither he nor his family were known to have hepatitis B infection and his AFP assay was normal. There was no evidence of disseminated disease despite the presence of a large neoplasm.

An ultrasound demonstrated an area of abnormal echotexture in the left lobe of the liver. The right lobe was normal. A C.T. scan was then performed in order to determine the exact nature of this lesion. It is important to realize that there are no

CT criteria which distinguish, absolutely, primary from secondary or benign from malignant neoplasms[8]. Although systemic contrast computer tomography may define individual lesions better there is no increase in the diagnostic yield[9]. This lesion was single, isodense and protruded from the contour of the liver. There were no focal areas of calcification such as are associated with haemangiomata[8]. Nor was there evidence of cirrhosis or central tumour necrosis as may be seen in patients with hepatomas[10]. Had bolus injection of contrast been performed, a centripetally advancing border of tissue enhancement might have been seen thus alerting us to the presence of a malignant tumour[10,11,12]. In our patient, C.T. scanning showed a large mass which enhanced after infusion of contrast. It did not provide us with a diagnosis.

The next investigation performed was a hepatic artery arteriogram. This demonstrated a hypervascular lesion in the left lobe of the liver with neovascularity, a tumour blush and AV shunting. The appearance was very suggestive of a malignant hepatic tumour. However, both benign and malignant tumours of the liver may show vascular enhancement during arteriography. Occasionally AV shunting may be seen in hepatic adenomas[14], hepatic regeneration[15] and cirrhosis[16]. A leiomyosarcoma reported by Baum[13] also showed enhancement and displacement of the intrahepatic arterial branches with irregular tumour vessels and a tumour blush late in the arterial phase.

Venous phase films in our patient showed occlusion of the left portal vein. This may occasionally be seen in healthy individuals[17]. Normally however, this finding would be considered a manifestation of an invasive tumour. Direct communication between the hepatic and portal venous systems is another radiological feature of tumour invasion and was also seen in this patient[17].

Prior to operating upon this patient the most likely diagnosis appeared to be a fibrolamellar hepatoma. These are rare variants of hepatocellular carcinomas. They frequently occur in adolescents and show no apparent sexual predisposition. The patients have no evidence of previous hepatitis B infection or cirrhosis and the AFP assay is normal. They are found more frequently in the left lobe of the liver rather than the right lobe[18]. Angiography of these lesions usually demonstrates a moderately vascular tumour as in this case[18].

Even though this was a large tumour an attempt at resection was justified in view of the patient's age and because fibrolamellar hepatomas have a longer mean survival and resection is recommended even if the tumour extends across the principal plane of the liver[19,20].

At operation the large mass in the left lobe of the liver was initially thought to be inoperable because of the way in which it appeared to infiltrate along the falciform ligament and because of the dense attachments between tumour and the inferior vena cava. The decision to proceed was finally made after frozen section histology became available and a diagnosis of leiomyoma was suggested. Even at this stage a leiomyosarcoma could not be excluded.

The diagnosis of leiomyoma fulfils the criteria outlined by Hawkins *et al.*[7]. The tumour was composed primarily of smooth muscle cells, there were no other leiomyomas found in the gastrointestinal tract and there were no histological features of malignancy.

It is fascinating that all four leiomyomas reported including this case, have arisen in the left lobe of the liver. One plausible site of origin may be vascular smooth muscle. Key and Raghunatha[21] have described a case of multiple tumours which they described as being haemangiomas with a prominent smooth muscle component. They confirmed the vascular origin of the tumours by immunoperoxidase staining for factor VIII - related antigen. This stain was not used in this case as the vessels within the tumour did not fit Key and Raghunatha's description of thin walled vascular spaces with tumour cells blending into the tunica media. Bile ducts are an unlikely origin as even large extrahepatic ducts have very few smooth muscle cells[22].

In view of the left sided distribution of these tumours it is possible they may arise from the falciform ligament but a careful search of the falciform ligament in this patient and three other patients failed to reveal any smooth muscle cells.

This case report emphasises the need for accurate diagnosis prior to deciding that a tumour in the liver is inoperable. Although this tumour was benign it would no doubt have eventually obstructed the inferior vena cava and perhaps even the portal vein. An aggressive surgical approach has led to the apparent cure of this patient although follow up at this time is short.

Previous papers about benign liver neoplasms have not emphasised the ability of benign lesions to masquerade as malignant ones. This possibility must always be considered as hepatic surgery may be curative.

### *References*

1. Ishtak, K.G. and Rabin, L. (1975). Benign tumors of the liver. *Medical Clinics of North America*, **59**(4), 995–1013.
2. Foster, J.H. (1977) Primary benign solid tumors of the liver. *American Journal of Surgery*, **133**, 536-541.
3. Foster, J.H. (1982) Benign liver tumors. *World Journal of Surgery*, **6**, 25-31.
4. Rovere, G.A della, Nigro, M., Besuzio, S. (1982) Primary tumours and tumour-like conditions of the liver. In *Liver Surgery*, edited by Roy Y. Calne, Padua: Piccin Medical Books Padua.
5. Demel, P. (1926) EM Operiorter Fall von Le bermyom; Virchows *Archives*, **261**, 881-4.
6. Rios-Dalenz, J.L. (1965) Leiomyoma of the liver. *Archives of Pathology*, **79**, 54-56.
7. Hawkins, E.P., Jordan, G.L., McGavran, M.H. (1980) Primary leiomyoma of the liver. *American Journal of Surgical Pathology*, **4**, 301-304.
8. Stanley, R.J. (1983) Liver and biliary tract. In *Computed Body Tomography*, edited by J.K.T. Lee., S.S. Sagel., R.J. Stanley, Chapter 7, pp. 167-211. New York: Raven Press.
9. Prando, A., Wallace, S., Bernandino, M.E., Lindell, M.M. (1980) Computed tomographic arteriography of the liver. *Radiology*, **130**, 697-701.
10. Itai, Y., Furui, S., Araki, T., Yashiro, N., Tasaka, A. (1980) Computed tomography of cavernous haemangioma of the liver. *Radiology*, **137**, 149-155.
11. Barnett, P.H., Zerhouni, E.A., White, R.I., Seigelman, S.S. (1980) Computed tomography in the diagnosis of cavernous haemangioma of the Liver. *American Journal of Radiology*, **134**, 439-447.
12. Johnson, E.H., Sheedy, A.F., Stanson, A.W., Stephano, D.H., Hattery, A.A., Adson, M.A. (1981) Computed Tomography and Angiography of Cavernous Haemangiomas of the Liver. *Radiology*, **138**, 115-121.
13. Baum, S. (1983) Hepatic arteriography. In *Abrams Angiography: Vascular and Interventional Radiology*, edited by H. L. Abrams, 3rd edn. Chapter 64, pp. 1479-1504. Boston: H.L. Brown and Co.
14. Goldstein, H.M., Neirnan, H.L., Meng, E., Bookstein, J.J., Appelman, H.D. (1974) Angiographic findings in benign liver cell tumours. *Radiology*, **110**, 339-43.
15. Farrell, R., Steinman, A., Green, W.H. (1972) Arteriovenous shunting in a regenerating liver simulating hepatoma. Report of a case. *Radiology*, **102**, 279-280.
16. Okuda, K., Moriyama, M., Yasumoto, M., *et al.* (1973) Roentgenologic demonstration of spontaneous reversal of portal blood flow in cirrhosis and primary carcinoma of the liver. *American Journal of Roentgenology*, **119**, 419-428.
17. Bergstrand, I. (1983) Splenoportography. In *Abrams Angiography*: *Vascular and Interventional Radiology*, edited by H.L. Abrams, 3rd edn. Chapter 67, pp. 1479-1504. Boston: Brown and Co.

18. Dawson, J.L. (1986). An unusual hepatic cancer, *British Journal of Surgery*, **73**, 331.
19. Soreide, O., Czemiak, A., Blumgart, L.H. (1985) Large hepatocellular cancers: hepatic resections or liver transplantation, *British Medical Journal*, **291**, 853-857.
20. Starzl, T.E., Iwatsuki, S., Shaw, M.A., Nalesnick, D.C., Farhi, D.C., Van Thiel, D.V. (1986) Treatment of fibrolamellar hepatoma with partial or total hepatectomy and transplantation of the liver. *Surgery of Gynecology and Obstetrics*, **162**, 145-148.
21. Key, R.D., Raghunatha, N.R. (1986) Leiomyomatous Hemangiomas of the liver mimicking primary leiomyomas. *Archives of Pathology Laboratory Medicine*, **110**, 858-859.
22. Mahour, G.H., Wakim, K.G., Soule, E.H., Ferris, D.O. (1967) The structure of the common bile duct in man: Presence or absence of smooth muscle cells. *Annals of Surgery*, **166**, 91-94.

*Accepted by S. Bengmark on 18 October 1988*

